# The role of quality institutions and technological innovations in environmental sustainability: Panel data analysis of BRI countries

**DOI:** 10.1371/journal.pone.0287543

**Published:** 2023-06-23

**Authors:** Xudong Gao, Mingjun Fan

**Affiliations:** 1 China Center for Special Economic Zone Research, Shenzhen University, Shenzhen, China; 2 Northeast Asian Studies College, Jilin University, Changchun, China; Hamdard University, PAKISTAN

## Abstract

The majority of countries struggle to accomplish sustainable development and environmental sustainability; nevertheless, environmental degradation issues can be resolved by enhancing technological innovations and institutional effectiveness. This study assesses the impact of technological innovations and institutional quality on carbon dioxide emission in the Belt and road initiative countries for the time period of 2002 to 2019. Fixed effect, OLS, and generalized method of moment estimators were applied to the panel data for analysis. The results shows that energy from fossil fuels, economic growth and technological innovations increase environmental degradation by rising carbon dioxide emission. Renewable energy consumption, the rule of law, and the quality of institutions make a significant contribution to the improvement of environmental quality. In particular, the Environmental Kuznets Curve and Innovation Claudia curve is valid in the Belt and Road Initiative countries. In the presence of quality institutions, countries can achieve sustainable growth and environmental sustainability by expanding their use of green technology and renewable energy. The findings provide suggestions to the sample countries on the improvement of institutional framework and technological innovations in order to achieve sustainable development.

## Introduction

Achieving sustainable development and environmental sustainability is a problem for the majority of countries, particularly developing and emerging countries that are concentrating on increasing social and economic growth. However, rising economic growth necessitated high energy consumption, leads to a rise in carbon discharge [1]. These countries mostly rely on the use of energy from fossils and not yet reached the level to fully utilize energy from renewables in order to combat the high carbon emission to safeguard environmental quality. Energy derived from fossil fuels increases carbon dioxide emissions and degrades environmental quality, whereas energy derived from renewable sources is ecologically benign, reduces carbon dioxide emissions, and enhances environmental quality. Carbon dioxide emissions have increased due to the increased use of fossil fuels, and a quick increase will be necessary to reduce emissions and achieve sustainable development targets (IEA, 2021) [2]. Environmental valuation can helps policymakers to include environmental factors in economic decision-making, promoting sustainable management of natural resources and enables society to understand the true value of the services provided by natural resources [3]. The developmental activities in countries leads to increased carbon emissions and multinational companies operating within the country worsen the situation of environmental quality [4]. The development of the economy grows which rise carbon emission however the country may still get closer to its goal of carbon neutrality through the growing use of renewable energy, technological innovation, and sustainable forest management [5, 6]. However it’s been also argued that sustainable growth can be achieved through the reduction of human activities that deplete the environment [7]. These studies used different factors that affect environmental quality positively or negatively based on the countries level however renewable energy use and technological innovations are considered important in reducing environmental degradation. Therefore, renewable energy and technological advances are required to achieve the United Nations’ environmental goals (United Nations, 2015) [8]. Additionally, it is difficult for these nations to acquire renewable energy sources; yet, a higher level of technological innovation could help these nations acquire renewable energy sources and improve energy efficiency [9]. By enhancing the energy sector and obtaining renewable energy sources, a development in green technology innovation will increase environmental quality [10, 11]. Innovation in green technology considerably improves environmental quality by contributing to a variety of economic activities and reducing pollutants. To increase energy efficiency and acquire renewable energy through technological innovation for utilization in economic activities, strong and dependable institutions may be required. Institutions with a high level of strength are able to enact policies pertaining to the improvement of technological advancements and improved environmental regulations for the protection of the environment [12]. In addition to establishing policies for economic activities related to environmental sustainability, institutions can also adopt policies for other economic activities. Thus, an augmented and strong level of institutional quality is advantageous for the expansion of economic activities and economic growth, as well as the establishment of laws to protect environmental quality [13].

This discussion demonstrates that for sustainable development and environmental sustainability, both institutional excellence and technological innovation are required. To determine the role of the Belt and Road countries in sustainable development, it is necessary to examine their institutional quality and level of technological innovation. Previous research on the sample countries has focused solely the role of institutional quality and technology innovations while energy from nonrenewable sources, renewable energy consumption and economic growth have not yet attempted. As stated previously, this association between technological innovation and improving energy efficiency, the acquisition of renewable energy sources, and the facilitation of economic activities with low carbon emissions is incomplete. Similarly, the quality of institutions can be related to technological innovation, and consequently, the quality of institutions can contribute to the enhancement of technological innovation. It is essential to explore the role of technological innovation and institutional quality in facilitating economic activities with low carbon emissions by increasing renewable energy sources and promoting industrial energy efficiency. It is necessary to investigate the role of institutions in formulating policies for technological innovations, economic activity, and environmental quality. Similarly, the basic direct effect of technological innovation has been researched in previous studies, but the Innovation Claudia Curve in the Belt and Road countries is being studied for the first time. This study also investigates the environmental Kuznets curve. By examining this association, sample countries will be able to receive incredibly pertinent policy advice for how Innovation Claudia and the Environmental Kuznets Curve can be obtained in the sample countries. The Innovation Claudia Curve indicates that if innovations are below the permissible level for enhancing environmental quality, carbon emissions will continue to rise even when there are more patented applications and innovation hits its peak. When countries reach their maximum degree of economic development, the Environmental Kuznets curve will determine whether economic expansion will continue to increase carbon dioxide. Thus, innovation Claudia and the environmental Kuznets curve are related, and it is crucial to determine the effect of technological innovation and economic growth on the carbon dioxide peak level. If these hypotheses are untrue, then Belt and Road countries must focus more on preventing poor development outcomes in the near future. This study found that technological improvements, the use of fossil fuels energy and economic growth degrade the environmental quality. On the other hand, rule of law, high quality institutions and renewable energy significantly rise environmental quality. Furthermore, the Innovation Claudia curve and environmental Kuznets curve is valid in the sample countries of Belt and Road initiative.

The remaining sections of the paper are organized as follows: Part 2 contains a review of pertinent prior literature to scrutinize the current gap; Part 3 presents the research methodologies and variables utilized for analysis; Part 4 presents the discussions and results; and Part 5 offers recommendations for the sample countries.

## Literature review

Several researchers in the preceding studies believe that an increase in technological innovations significantly raises the amount of carbon dioxide in the atmosphere and causes a decline in the quality of the environment, contrary to the findings of some researchers Albino, Ardito et al. (2014); Raiser, Naims et al. (2017) [14, 15]. It’s possible that countries with different rates of economic growth, innovation, and quality of institutions will experience this influence differently. It has been hypothesized that technological advancement will supply sources of renewable energy, which will, in turn, improve environmental quality by lowering carbon dioxide emissions [16]. Obobisa, Chen et al. [17] examined the effect of green technology innovations, renewable energy, institutional quality, and economic growth on carbon dioxide emission from 2000 to 2018 in 25 African countries. Using cross sectional, heterogeneity and AMG tools, the findings shows that green technology innovations and renewable energy consumption negatively affect carbon dioxide emission. The effect of economic growth, fossil fuels and institutional quality on emission is positive which reduce environmental quality. Likewise, Wenlong, Tien et al. [18] explore the effect of technological innovations, quality institutions and energy efficiency on greenhouse gas emission in Asian countries from 1995 to 2018 using augmented ARDL, AMG and CCEMG. The findings shows that institutional quality have a detrimental impact whereas technological innovation and energy efficiency have favorable effect on environmental quality. Chien, Ajaz et al. [19] studied carbon dioxide emission reductions in Pakistan, together with the effects of technological advancement and the utilization of renewable energy sources. The author conducted an analysis of data spanning from 1980 to 2018 to demonstrate that advancements in technology and the use of renewable energy contributed to a reduction in the amount of carbon dioxide emitted. In Pakistan, the authors provided confirmation of the environmental Kuznets curve. Other studies have stated that technological developments will improve renewable energy, which is consistent with the findings of this author who found that the usage of renewable energy reduced emissions of carbon dioxide. Therefore, technological innovation and the use of energy sources that do not deplete the earth’s natural resources are essential for sustainable growth. Ali, Jianguo et al. [20] investigated the effect of economic policy and financial development on carbon emission followed by institutional quality, technological innovation, energy consumption, economic growth and FDI in OECD countries from 2003 to 2019. Using second generation methods include two stage sequential methods and GMM model. The findings shows that economic policy uncertainty, financial development, economic growth and energy consumption lowers the quality of environmental however institutional quality and technological innovations reduce emission. Chhabra, Giri et al. [21] studied the effect of institutional quality and trade openness on carbon dioxide emission from 1991 to 2019 in BRICS countries. Using dynamic common correlated effects model, the findings shows that trade cause environmental degradation while institutional quality significantly reduce emission and raise environmental quality. Similarly, Ozkan, Khan et al. [22] explore the effect of green technological innovations on turkey environmental quality using dynamic ARDL model. The results shows that green technology innovations reduce ecological footprint in Turkey and raise environmental quality. Shan, Genç et al. [23] investigated the significance of the use of renewable energy and the development of environmentally friendly technology in Turkey’s effort to achieve carbon neutrality. The STIRPAT model was applied to data spanning the years 1990 to 2018, during which time renewable energy and technological innovation made significant strides in reducing carbon dioxide emissions; however, economic growth per capita, energy consumption, and population growth all contributed to an increase in carbon dioxide emissions. Despite this, Sharma, Shahbaz et al. [24] examined the impact that advances in technology have had on the utilization of renewable energy sources in the BRICS countries. The authors conclude, using data ranging from 1990 to 2018, that developments in technology help speed up the development of cleaner energy options. Suki, Suki et al. [25] conducted research to investigate the impact that renewable energy and technology have had on Malaysia’s efforts to cut carbon dioxide emissions. The findings, analyzed with the BARDL model, demonstrate that the use of renewable energy sources leads in lower levels of carbon dioxide emissions. Additionally, advancements in technology lead to lower levels of carbon dioxide emissions, which improves the quality of the environment. Bilal, Li et al. [26] investigated the relationship between green technology innovation, globalization, and carbon dioxide emissions in the nations that make up the belt and road. Using methods of the second generation, the authors analyzed data spanning the years 1991 to 2019. The authors found a negative link between carbon dioxide emissions and technological innovation in all regions, including South Asia, South East Asia, West Asia, Central Europe, the Middle East, and North Africa. Consumption of energy and overall economic expansion are two of the many other elements that have been identified as contributing factors to overall carbon dioxide emissions. In the study, Cheng and Hou [27] investigate the connection between technological innovation and economic expansion in 48 different countries. The scientists looked at data spanning from 1971 to 2015 and discovered that the relationship between economic growth, financial innovation, and innovation in general varies depending on the time period studied as well as the socioeconomic level of the population. Studies have been done looking at how carbon dioxide levels, technological innovation, and institutional quality all relate to one another Bakhsh, Yin et al. [28]. The authors examined data from forty different Asian nations between the years 1996 and 2016, and they came to the conclusion that institutional quality moderates the link between carbon dioxide and foreign direct investment, and that technological innovation is essential. In addition, the authors provide evidence that increased levels of foreign direct investment and high-quality institutions considerably slow the pace of environmental degradation. Abid, Mehmood et al. [29] investigated the relationship between carbon dioxide levels, the advancement of technology, and the consumption of energy. For this study, data samples from G8 countries were used from 1990 all the way up till 2019. They discovered that the long-term association between carbon dioxide levels and technological innovation was negatively correlated using FMOLS econometric models. The author presents supplementary evidence to support the claim that urbanization diminishes environmental quality. In a different piece of research Zhang [30], the author explores the relationship between technological advancement, increased carbon dioxide levels, and increased economic growth. For the BRICS countries, samples of data spanning the years 1990 to 2019 were compiled. In the course of their investigation, which was guided by the STIRPAT model, they came to the conclusion that advances in technology bring about a large cut in emissions. On the other hand, they found that an increase in economic growth leads to an increase in carbon dioxide emissions. When the square term of economic growth is considered, the environmental Kuznets curve theory is only supported for two of the sample’s economies. Another researcher analyzed the BRICS sample to determine the relationship between technological advancement, economic growth, and carbon dioxide emissions Shen, Su et al. [31]. Following an investigation into the EKC hypothesis, the author came to the conclusion that innovation indicators lead to an increase in carbon dioxide emissions, while innovation proxies lead to a decrease in emissions. In a similar vein, mobile cellular subscriptions provide a substantial benefit to the environment. According to the findings of this study, additional factors that contribute to carbon dioxide emissions include high-tech exports, international trade, and energy use. Researchers looked into the connection between advances in technology and rising levels of carbon dioxide [32]. The research was conducted from 2009 to 2018, and it includes a sample from each of China’s thirty provinces. They used a model with a fixed impact and came to the conclusion that emissions from the technology innovation curve and an increase in technological innovation contribute to China’s sustainable growth [33] examined the association between innovation, technological advancement, and emission levels between the years 2000 and 2019 in a variety of nations with varied levels of economic growth. They used the DOLS and FMOLS models, and found that patents and R&D lead to lower levels of carbon emissions. Li and Wei [34] conducted research that examined innovation, economic growth, and carbon dioxide emissions from 1987 to 2017. The provinces of China served as the research project’s sample population. The developers of panel-based econometric models discovered a nonlinear connection between the several variables that were being looked into. However, the authors also observe that different levels of carbon dioxide had a variety of effects on economic growth for the different subsamples. By employing the ARDL estimator, Fan and Hossain [35] investigated the relationship between technological innovation, economic growth, and carbon dioxide levels in China and India. The study looked at data from 1974 to 2016, and its findings suggest that technological innovation and increased carbon dioxide levels contribute to sustained economic growth. They came to the conclusion that this effect was inconclusive in the short run. In a similar vein, they found that the long-term impact of carbon dioxide on growth was good, whereas its impact on growth in the near term was detrimental. Large number of studies that using other factors and variables which can be linked to environmental quality have also been established for different time period and samples such as Adebanjo and Shakiru [36] investigate the dynamic linkage between air pollution and economic growth in Jordan. Using cointegration methos, the findings shows that there exist long term association between air pollution and economic growth. The findings further shows that the EKC hypothesis was valid. Jamil, Rasheed et al. [37] explore corporate social responsibility impact on organization sustainable growth in Pakistan using data of 196 listed firms in Pakistan stock exchange. Using OLS regression, the findings shows that highly significant reliable, and sustainable. Sustainable Corporate Social responsibility is the leading factor that enhances the firm performance. Firm size and age are significant for sustainable organizational growth (firm performance). Adebanjo and Adeoye [38] studies global initiatives in the face of natural resources depletion in sub Saharan Africa. Applying Hausman test and fixed panel regression model, the results shows that transparency indicators that includes voice and accountability, and corruption have a positive significant impact on the economic growth of the 10 SSA countries under consideration, indicating that transparency is a critical factor in determining good economic performance. Jamil [39] studies energy consumption and its impact on economic growth in income level countries from 1971 to 2021. Employing cointegration and VECM model, the findings shows that Oil, Gas and Electricity are equally important short run/long run, while the coal log-run is more than in the short-run. The energy consumption to economic growth has a unidirectional causality, indicating energy is a factor that affects country growth. Regression results also confirm that energy significance on top for economic growth, Energy Sources; Gas and Electricity were useful but energy source Oil getting more attention in past decades. Banerjee [40] studied FDI flow in energy sectors in Asian and sub regional alignments. A major chunk of commerce is aided by a strong relationship between service trade and foreign direct investment flow between countries or regions. South East Asian ASEAN countries played an essential role in attracting foreign direct investments for economic development and expansion in the energy sector. Trade obstacles, particularly commitment barriers imposed by importing countries, have a significant negative influence on the free flow of investment between regions. This study also examines the goals of foreign direct investment among regional countries and investigates the most deep and strong links among regional members in order to investigate a possible strategic relationship for the development of a Regional Trade Agreement. Modi, Isyaku et al. [41] including a courtyard as a building component is one of the most environmentally friendly ways to improve the thermal performance and microclimate of a structure. This study studied the effect of direction on the thermal performance of the fully enclosed courtyard while using the Envi-met program on the configurations chosen for the fully enclosed courtyard. Raihan [42] studied environmental Kuznets curve and pollution haven hypothesis in Bangladesh from 1990 to 2019 using ARDL model. The findings shows that the EKC is valid and pollution haven is also existed in Bangladesh. Chukwuma, Ugwu et al. [43] studied forensic accounting in predicting the financial performance growth of MTN mobile communication in Nigeria. The authors used world bank publication data, Nigeria stock exchange factbook and national bureau of statistics record from 1990 to 2021. OLS, and cointegration methods were used and found that there is a significant relationship between forensic accounting instruments and economic growth in financial performance. Robeena Bibi [44] examined the relationship between trade openness, economic growth, and financial development in four countries of south Asia from 1980 to 2017 using dynamic panel methods. The results shows that stock market development positively affect economic growth in the sample countries. Bibi explores the effect of banking sector growth on economic growth in south Asian countries from 1980 to 2017. The findings show that bank-based index significantly and positively affect economic growth in the sample countries. BiBi studied the effect of FDI on economic growth in global income economies from 1998 to 2018 using dynamic panel models. The findings shows that FDI positively affect economic growth in the global panel, lower middle, and upper middle-income countries where it was not valid for high income countries. The effect of banking sector growth effect economic growth negatively and significantly in global panel, high income and upper middle-income economies. The effect of stock market on economic growth in global panel was also negative in this study. Jamil [45] studied monetary policy performance under control of exchange rate and consumer price index from 1955 to 2021 using generalized method of moment estimator. The findings shows that monetary policy control, the price level does not affect exchange rate and production however monetary policy can change the trend of consumer price index and exchange rate. Jamil [46] explore the impact of choice of exchange rate regime on country economic growth from 1961 to 2020. Using GMM model and found that post Bretton woods transition from fixed to flexible management, strong association exist among the choice of exchange rate regime and countries growth. Bibi [47] studied financial development and economic growth in south Asian countries using dynamic panel and static models. The findings shows that both stock market and banking sector increase economic growth in the sample countries. Cristobal-Cipriano, Arroyo et al. [48] studied the corporate social responsibility program by business organization in Sarangani province. The findings shows that CSR drivers were customer satisfaction and the company reputation while the major barrier was the lack of top management support. Bakare and Okuonghae [49] explore the role of information managers in accomplishing sustainable goals in Nigeria in 21th century. The study shows that libraries are vital instruments in society and they play a critical role in accomplishing long term sustainable development goals. Al-kasasbeh [50] studied the macroeconomic impacts and understand its implication for Jordan in the Covid-19 pandemic era. The authors show that the economic effects of the COVID-19 have been highlighted in this study and emphasized policy options to reduce its effects. The study comes to the conclusion that monetary, macroprudential, and fiscal policy can help mitigate the effects of the COVID-19. YANOS [51] studies rainfall variability in the Quirino provinces from 1997 to 2016. The annual series was used to screen each station annual rainfall data and found that to attain allowable error in estimation of 10%, 5% and 1% for the mean annual rainfall the number of rain gauge station needed in the province should be 18, 72 and 1799, respectively.

## Methodology

### Empirical models and variables

This study evaluates the impact of green technology innovation and quality institutions on carbon dioxide emissions in the belt and road initiative countries from 2002 to 2019. In addition to economic growth, renewable energy consumption, and fossil fuel energy, more closely related variables are added to the model. The innovation and economic growth square term is introduced to the model in order to examine the green technology innovation Claudia and environmental Kuznets curves. According to the Claudia curve idea, carbon dioxide emissions will grow initially due to restricted accessibility, but will decrease as more inventions get patented. The EKC theory demonstrates that economic expansion increases carbon dioxide during the first stage of development, whereas environmental quality increases during subsequent phases. In the approach, institutional quality is proxied by the rule of law, and the innovation index is proxied by three legal system indicators, including voice and accountability, regulatory quality, and the rule of law. This index is used by Khan, Weili et al. (2021) [52]. Innovations in green technology are seen as crucial for improving environmental quality Sun, Edziah et al. (2019) [53]. Several proxies have been used to proxy technological innovation in past research, but patents have become the most prevalent proxy recently Youssef, Boubaker et al. (2018) [54].

Similarly, institutional quality increases the quality of the environment, according to a significant number of researchers such as Khattak, Ahmad, Khan, & Khan, (2020) [55]. Moreover, some researchers have found that a rise in economic growth causes an increase in carbon dioxide emissions and a decline in environmental quality, such as Gorus and Aslan (2019) [56], and that this beneficial influence of economic expansion on carbon emissions is due to the high amount of energy use Aust, Morais et al. (2020) [57]. The use of renewable energy as a substitute for energy from nonrenewable energy sources is regarded as good to environmental quality Khan et al. (2021) [58]. Eqs 1 and 2 show how the variables in the study are related to each other in the linear and nonlinear baseline models of the study.


$${CO2}_{it}=\beta_{0}+\beta_{1}{GTINV}_{it}+\beta_{2}{GDP}_{it}+\beta_{3}{RE}_{it}+\beta_{4}{FSF}_{it}+\beta_{5}{INST}_{it}+{ɛ}_{it}\ldots \ldots \ldots \ldots \ldots \ldots .$$ (1)

$${CO2}_{it}=\beta_{0}+\beta_{1}{GTINV}_{it}+\beta_{2}{GTINV}_{it}^{2}+\beta_{3}{GDP}_{it}+\beta_{4}{GDP}_{it}^{2}+\beta_{5}{RE}_{it}+\beta_{6}{FSF}_{it}+\beta_{7}{INST}_{it}+{ɛ}_{it}\ldots \ldots \ldots \ldots \ldots \ldots .$$ (2)

CO2 stands for carbon dioxide emissions in metric tons per capita, GDP stands for economic development as defined by gross domestic product per capita, RE stands for consumption of renewable energy, FSF stands for consumption of energy derived from fossil fuels, and INST stands for institutional quality. In Table 1, the variables of the investigation are presented, while in Tables 2 and 3 shows the descriptive statistics and correlation matrices for these variables are shown.

**Table 1:** Variables description.

Variables	Symbols	Description
**Carbon dioxide**	CO2	Carbon dioxide emission (metric tons per capita)
**Green Innovation**	PAR	Patent application (residents)
**Institutions**	INST	Rule of law
**Renewable energy consumption**	RE	% of total final energy
**Fossil fuels energy consumption**	FSF	% total energy consumption
**Economic growth**	GDP	Per capita gross domestic product
**Voice and accountability**	VOA	Voice and accountability
**Regulatory quality**	RQ	Regulatory quality
**Rule of law**	ROL	Rule of law

**Table 2:** Descriptive statistics.

Variable	Mean	Std. Dev.	Min	Max
**CO2**	4.481	3.421	0.001	15.047
**PAR**	17457.34	120433.2	1.000	14000
**GDP**	3.984	3.818	-14.759	17.031
**RE**	24.217	22.157	0.001	93.456
**FSF**	74.831	19.530	0.002	99.977
**VOA**	-0.200	0.862	-1.829	1.213
**RQ**	0.045	0.752	-1.720	1.698
**ROL**	-0.123	0.670	-1.371	1.3728

**Table 3:** Correlation matrix.

	**CO2**	**PAR**	**GDP**	**RE**	**FSF**	**VOA**	**RQ**	**ROL**
**CO2**	1.000							
**PAR**	0.085	1.000						
**GDP**	-0.059	0.125	1.000					
**RE**	-0.580	-0.069	0.029	1.000				
**FSF**	0.241	0.103	-0.025	-0.744	1.000			
**VOA**	0.298	-0.244	-0.133	-0.043	-0.251	1.000		
**RQ**	0.372	-0.079	-0.086	-0.102	-0.210	0.866	1.000	
ROL	0.392	-0.091	-0.149	-0.109	-0.155	0.838	0.909	1.000

### Econometric models

Before performing any kind of formal analysis, the stationarity of the data for each variable is determined. The CIPS and CADF tests of the second generation were used to assess the stationarity of the data. These tests assume that each series has an independent distribution over the cross-sectional areas of the panel. These tests depend on the delay value of the mean cross-sectional and the single series of the first difference to enhance the ADF regression. The elimination of common components identified by cross-sectional correlation and the provision of the homogeneity null hypothesis for each panel and region. Consequently, testing against a variety of alternatives that consider regional and national variances. The conventional least squares method, fixed effects modeling, and the extended method of moments are employed as modeling techniques (GMM). The static models are employed for comparison purposes, but the GMM estimator, and more specifically the two-step system GMM, which is commonly regarded as the most precise estimator, is the primary focus of this work. The implementation of static models such as OLS, which are afflicted by a number of econometric problems such as autocorrelations, would result in the creation of inaccurate findings. It is possible that the OLS estimator and the fixed effect model will confront comparable econometric obstacles. Due to the possibility of a time-invariant fixed effect correlation between explanatory and dependent variables in the error term, the fixed effect model IV estimator instruments may yield inefficient and erroneous results, and they may also be biased similarly to the OLS model. In turn, the GMM model gives the most reliable findings for panel data analysis Weili, Khan et al. [59]. There are two types of GMM estimators, including difference GMM models and system GMM models. The most popular type is the difference GMM model. The GMM model utilizes the first difference between the dependent and independent variables and instruments the first difference lag dependent variable using its past values. This is done in order to lessen the effects that are unique to each nation. In such a scenario, the autocorrelation problem is resolved; nevertheless, when applying the first difference, the lagging levels may be deemed unreliable instruments, so diminishing their value. The system GMM estimator was created by Arellano and Bover (1995) [60] and Blundell and Bond (1998) [61] to boost the model’s efficiency. The system GMM estimator is considered more effective than the difference GMM model. Two equations are utilized to simulate a system with GMM. The initial equation is a differential equation, while the second equation is a linear equation. Both of these equations are not linear KURUL, (2021) [62]. After that, the second variable in the equation is transformed to its first-order difference, and the lag level of the variable is determined based on how it influences the difference. Arellano and Bover (1995) [60] discovered that the generalized technique of moments was effective for analyzing panel data when there was a small T period and a large N (number of countries) in the sample (Arellano and Bover, 1995) [60]; (Blundell & Bond, 1998) [61]. The results of the GMM model, especially the most recent two-step GMM model, will receive the most attention from this study; however, the study also includes both static and dynamic models, including fixed effect, OLS, and GMM.

## Results and discussions

In this section, the findings and interpretations of the research are discussed. Before beginning the more in-depth investigation, the data were put through a series of second-generation panel unit root tests to ensure that they were in a stationary state. We go on to formal analysis using static and panel data models since the panel unit root of the second-generation CIPS and CADF reveals that the data for all variables is stationary in level or in first difference. The results of panel unit root tests are presented in Table 4. As a result, we move on to formal analysis using these models. Due to the fact that these models have been determined to be suitable for panel data analysis, the results of the dynamic models that have been given in the methodology section will be the primary focus of the investigation.

**Table 4:** Panel unit root tests.

Variables	CIPS		CADF	
	I(0)	I(1)	I(0)	I(1)
**CO2**	-1.540	-4.670***	-1.302	-2.764***
**PAR**	-2.245***	-4.222***	-2.236***	-3.376***
**GDP**	-2.734**	-4.345***	-2.062	-2.712***
**RE**	-0.582	-3.987***	-0.183	-2.105***
**FSF**	-0.928	-3.281***	-0.808	-2.170***
**VOA**	-1.833	-3.533 ***	-1.875	-2.807 ***
**RQ**	-2.291	-4.241***	-1.726	-2.436***
**ROL**	-2.256	-4.010***	-2.089	-3.002***

Table 5 present the results on the effect of green innovation and institutions on carbon dioxide emission. The results indicate that the carbon dioxide lagged dependent variable is very significant and positively skewed, and the AR1, AR2, and Sargan tests validate the relevance of the model as well as the dependability of the instruments.

**Table 5:** Effect of green innovation and institutions on carbon dioxide emission.

VARIABLES	OLS	FE	DGMM	SGMM	SGMM
**GINV**	5.040***	5.010***	2.470***	3.100***	-1.640***
	(6.710)	(6.710)	(2.120)	(8.120)	(3.401)
**ROL**	0.349*	0.205	0.052***	-0.012***	
	(0.197)	(0.205)	(0.001)	(0.001)	
**INST index**					-0.021***
					(0.0005)
**GDP**	-0.000	-0.001	0.019***	0.041***	0.039***
	(0.007)	(0.007)	(0.000)	(0.000)	(0.0001)
**RE**	-0.023***	-0.019***	-0.008***	-0.000***	-0.0008***
	(0.006)	(0.006)	(0.000)	(0.000)	(0.0001)
**FSF**	0.049***	0.053***	0.043***	0.990**	0.001***
	(0.008)	(0.008)	(0.000)	(0.002)	(0.0003)
**L.CO2**			0.426***	0.989***	0.982***
			(0.001)	(0.003)	(0.0006)
**Constant**	1.691**	1.438*		-0.039	-0.113***
	(0.857)	(0.734)		(0.165)	(0.033)
**Observations**	434	434	351	400	627
**R-squared**		0.231			
**Number of id**	36	36	35	36	37
**AR1**			-1.60 (0.109)	-1.71 (0.086)	-1.92 (0.055)
**AR2**			-0.72 (0.472)	-0.66 (0.512)	-0.83 (0.407)
**Sargan Test**			307.21 (0.032)	292.24 (0.806)	455.6 (0.967)

Note: Standard errors in parentheses,

*** p<0.01,

** p<0.05,

* p<0.1

The most important variable is green technological innovation, whose coefficient is highly significant and sign is positive across most estimators, showing that advances in technological innovations in the sample countries increase carbon dioxide emissions. Our results are supported by H. Khan, Khan, and BiBi [62] who also discovered that technological innovation increases carbon dioxide however, Weili, Bibi et al. (2022) [63] argues that carbon dioxide and economic growth increase technological innovations while the inflow of FDI decrease innovations output where energy consumption also negatively affects innovation indicators. Which can support our findings up to some extent that the effect of technological innovations on emission is positive in our study which can be the reason of economic growth influence on technological innovation that worsen environmental quality. The results of this study imply that green innovation technologies in the sample countries have not yet achieved the level intended to cut carbon dioxide emissions and enhance environmental quality. This is also tied to other areas, such as the energy industry, because a rise in energy use results in a rise in carbon dioxide emissions. However, technological advances can cut energy use, enhance energy efficiency, and acquire energy from renewable energy sources, hence lowering carbon dioxide emissions. The findings of this study, however, imply that methods to improve energy efficiency and environmental quality have not yet been improved.

In contrast, we use an institutional quality index consisted of three institutional variables in column 5; as a result, the innovation coefficient is substantial and negative and can be connected with the level of institutions in the sample countries. These results indicate that institutional quality promotes technological innovation and, by extension, environmental quality. The findings of this study are consistent with those of Khattak, Ahmad et al. (2020) [64]. Additionally, these authors identified a positive correlation between technological innovation and carbon dioxide emissions. Our results, in contrast, contradict those of other studies of Suki, Suki et al. (2022) [65]; Youssef, Boubaker et al. (2018) [66] Our findings indicate that technological innovation in the sampled countries necessitates growth.

The rule of law is used as a proxy for the quality of institutions, with a substantial and negative coefficient in our two-step GMM target estimator. This finding implies that the quality of institutions (the rule of law) significantly enhances environmental quality where technological innovation and carbon emissions. Specifically, a 1% improvement in institutional quality in the countries of the belt and road will significantly reduce carbon dioxide emissions and raise environmental quality by 0.012%. The two-step GMM estimator employing the institutional quality index consisting of the three indicators stated in the methodology section yielded comparable results. The coefficient of the institutional quality index has a negative sign and is likewise extremely significant, corroborating the conclusions of a single indicator. The results are compatible with Azam, Liu et al. (2021) [67] and contradict Hassan, Khan et al. (2020) [68]. Consequently, our findings illustrate the importance of institutional quality in the Belt and Road countries’ attempts to reduce carbon emissions.

The effect of economic growth on carbon dioxide emissions demonstrates that the coefficient in the GMM estimator for a single proxy and the institutional quality index model is positive and significant, indicating that economic growth in the belt and road countries is responsible for high carbon emissions and environmental degradation. According to the results of models 1 and 2, the coefficients of the two-step GMM system imply that a one percent rise in economic growth per capita will increase carbon dioxide emissions by 0.041% and 0.039%. The positive impact of economic growth on carbon emissions may be the result of increased social and economic growth, as well as an increase in energy-intensive economic activities, such as industrial output. These countries, however, are not yet developed and lack the capacity to employ renewable energy sources; as a result, they rely on carbon-emitting fossil fuels for economic activities. Similarly, the level of innovation in the country has not yet been increased to promote energy efficiency. Currently, institutional quality contributes insufficiently to environmental preservation and other economic activities. Therefore, economic growth in the studied countries causes environmental damage. Our results are like those of Adams and Nsiah (2019) [69]; Dauda, Long et al. (2021) [70]. Khoshnevis Yazdi and Shakouri (2017) [71], on the other hand, discovered contradictory evidence that economic growth has a negative impact on carbon dioxide emissions. Zoundi (2017) [72] discovered similar findings, arguing that economic growth causes environmental degradation.

According to the corresponding equations, the effect of renewable energy on carbon dioxide is negative. According to the research, a rise in renewable energy reduces carbon dioxide emissions and enhances environmental quality. Although the anticipated coefficient of fossil fuel energy consumption is positive and statistically significant, this indicates that fossil fuel energy consumption degrades the quality of the environment. Compared to nonrenewable energy derived from fossil fuels, renewable energy has a favorable effect on environmental quality, according to the research. However, the countries along the Belt and Road may not yet have access to renewable energy for economic activity and may continue to rely on energy derived from environmentally dangerous fossil fuels. This may explain the technological innovation capacity of the sample countries. If these countries possess cutting-edge technology and innovative prowess, they may be able to generate renewable energy, thereby improving environmental quality. The findings suggest that in order to improve environmental quality, the sample countries should prioritize technological innovation to get renewable energy and discourage the use of fossil fuels. By replacing fossil fuels with technology and renewable energy, economic growth can be increased, and this will lead to sustainable development. Similar findings by Dauda, Long et al. (2021) [70]; (Suki et al., 2022) [65] confirm our conclusion that renewable energy reduces carbon dioxide. Similarly, Appiah, Du et al. (2019) [73] bolster our conclusion that the use of fossil fuels degrades environmental quality. Zhang, Wang, et al. (2017) [74] and Khan, Han, et al. (2021) [75] also concluded that using renewable energy sources reduces CO2 emissions. These authors present evidence that using renewable energy sources improves environmental quality while also lowering carbon dioxide emissions.

Table 6 explores the green technology innovation Claudia curve and the environmental Kuznets curve by inserting a quadratic term for technological advances and economic growth. Similar to the results of the previous table, the coefficient of technological innovation indicates that innovation is highly significant, and the sign is overwhelmingly positive for the vast majority of estimators, indicating that innovations in the sample countries increase carbon dioxide emissions. These results are inline with the findings of Demircan Çakar, Gedikli et al. (2021); [76] who also found that technological innovations increase carbon dioxide emission and leads to environmental degradation while Suki, Suki et al. (2022) [65] statutes that technological innovations reduce emission in Malaysia which contradict our findings. The results indicate that green innovation technologies in the sample countries have not yet reached the targeted level for reducing carbon dioxide emissions and improving environmental quality. This is also connected to other industries, such as the energy sector, because an increase in energy use leads to an increase in carbon dioxide emissions. However, technological advancements can reduce energy use, improve energy efficiency, and acquire energy from renewable sources, hence reducing carbon dioxide emissions. However, the results of this study indicate that strategies to improve energy efficiency and environmental quality have not yet been enhanced.

**Table 6:** Innovation Claudia and environmental Kuznets curve analysis.

VARIABLES	OLS	FE	DGMM	SGMM	SGMM
**GINV**	8.730***	8.700***	0.001**	7.470***	6.650***
	(1.410)	(1.390)	(0.001)	(3.930)	(1.8809)
**GINV** ^**2**^	-0.001***	-0.001***	-0.001***	-0.0001***	-0.001***
	(0.001)	(0.001)	(0.001)	(0.0001)	(0.001)
**ROL**	0.299*	0.188	0.192***	-0.490***	
	(0.158)	(0.161)	(0.002)	(0.002)	
**INST**					-0.001***
					(0.002)
**GDP**	0.007***	0.007***	0.127***	0.001***	0.001***
	(0.007)	(0.007)	(0.0001)	(0.000)	(0.001)
**GDP** ^**2**^	-0.001*	-0.001**	-0.009***	-0.018***	-0.001***
	(0.0008)	(0.0008)	(1.170)	(5.410)	(7.070)
**RE**	-0.023***	-0.019***	-0.008***	-0.000***	-0.014***
	(0.006)	(0.006)	(0.000)	(0.000)	(4.060)
**FSF**	0.032***	0.032***	0.070***	0.0001***	0.001***
	(0.003)	(0.003)	(0.000)	(0.0001)	(2.330)
**L.co2**			0.778***	0.001***	1.026***
			(0.0006)	(0.001)	(0.0002)
**Constant**	2.184***	2.110***		0.001***	0.001***
	(0.516)	(0.317)		(0.001)	(0.002)
**Observations**	666	666	592	629	627
**R-squared**		0.214			
**Number of id**	37	37	37	37	37
**AR1**			-3.27 (0.001)	-3.02 (0.003)	-2.00 (0.045)
**AR2**			-0.43 (0.669)	-0.26 (0.795)	-0.80 (0.424)
**Sargan test**			463.47 (0.511)	419.09 (0.999)	463.00 (0.937)

Note: Standard errors in parentheses,

*** p<0.01,

** p<0.05,

* p<0.1

The positive sign of the coefficient of the square term of innovations in green technology demonstrates that the innovation Claudia curve is applicable to the belt and road imitative countries. Khan, Khan et al. (2022) [77] found similar results for OECD countries that innovations positively effect carbon dioxide emission in the initial stage while reduce emission when innovation reach a certain level. Thus, their findings support our results. The fact that the innovation initially increases carbon dioxide emissions but then decreases them after it reaches its maximum level illustrates that the innovation has a substantial impact on environmental quality when additional applications are patented and technology is deployed.

In our two-step GMM target estimator, the rule of law serves as a proxy for the quality of institutions, with a considerable and negative coefficient. This conclusion suggests that the strength of institutions (the rule of law) substantially improves environmental quality at the intersection of technological innovation and carbon emissions. In particular, a 1% improvement in institutional quality in the belt and road countries will significantly reduce carbon dioxide emissions and enhance environmental quality by 0.012%. The two-step GMM estimator using the institutional quality index comprised of the three indicators outlined in the methodology section produced equivalent findings. The coefficient of the index of institutional quality has a negative sign and is also quite significant, confirming the findings of a single indicator.

The effect of economic growth on carbon dioxide emissions indicates that the coefficient in the GMM estimator for a single proxy and the institutional quality index model is positive and statistically significant, indicating that economic growth in the belt and road countries is accountable for high carbon emissions and environmental degradation. According to models 1 and 2, a one percent increase in economic growth per capita will result in a 0.001% rise in carbon dioxide emissions. This result is in line with Zoundi (2017) [72]; Chien et al. 2021; [19]. (Usman et al. 2020) [78] also found that economic growth exerts upward pressure on ecological footprint. However, our results are contradictory with Khoshnevis Yazdi and Shakouri (2017) [71]. The model incorporates the economic growth square term for Environmental Kuznets Curve testing. The calculated coefficient of its square term is highly significant and negative, confirming the presence of the Environmental Kuznets curve in the belt and road countries. Khan, Khan et al. (2022) contradict our findings whose study does not validate the Environmental Kuznets Curve in OECD countries. When countries achieve a particular degree of development, economic expansion greatly improves the quality of the environment by reducing carbon dioxide emissions; nevertheless, economic expansion causes environmental damage in the early stages of development. In terms of institutional quality and renewable energy utilization, our findings corroborate both the environmental Kuznets curve and the Innovation Claudia curve. The findings suggest that innovations are required to achieve sustainable development in the belt and road countries by boosting economic growth, energy efficiency, and hence sustainable development.

The estimated coefficient of renewable energy consumption is negative and statistically significant, indicating that the growth in renewable energy consumption in "Belt and Road" countries has significantly enhanced environmental quality. (Zhang et al. 2017a) [74] also found that renewable energy consumption reduces carbon emissions. The fossil fuel energy consumption coefficient is large and positive, indicating that fossil fuel energy consumption and fuel energy use will degrade environmental quality. In addition, the data indicate that the use of renewable energy is conducive to enhancing environmental quality, but the use of energy derived from fossil fuels can severely harm environmental quality. The Belt and Road countries may not yet have access to renewable energy for economic activities and may continue to rely on energy sourced from environmentally hazardous fossil fuels. This could explain the technological innovation capacity of the sampled countries. If these countries possess cutting-edge technology and inventiveness, they may be able to generate renewable energy, thereby enhancing environmental quality. The findings suggest that, in order to improve environmental quality, the countries in the sample should prioritize technological innovation to get renewable energy and discourage the use of fossil fuels. By replacing fossil fuels with technology and renewable energy, it is possible to improve economic growth, which will result in sustainable development. Dauda et al. (2021) [70] observed similar results, which supports our conclusion that renewable energy reduces carbon dioxide. Similarly, Appiah, Du, Yeboah, & Appiah, (2019) [73] reinforce our conclusion that the usage of fossil fuels degrades the quality of the environment.

## Conclusion

This study examines the influence that innovations in environmentally friendly technology and high-quality institutions had on the levels of carbon dioxide emissions produced by Belt and Road initiative countries between the years 2002 and 2019. During the analysis, models such as OLS, fixed effect, two-step difference GMM, and two-step system GMM were applied. The sample countries of the Belt and Road are considered as developing and emerging economies. These nations are placing a strong emphasis on broadening their economic activities in order to encourage both economic and social advancement. The economic activities of Belt and Road Initiative countries include international trade, foreign direct investment, etc. In contrast, industrial manufacturing is the dominant economic activity in these countries, which raises energy demand, carbon dioxide emissions, and environmental damage. As the countries are still at a low level, it is probable that they have not yet attained enough renewable energy to sustain economic activities and minimize pollution. To solve environmental concerns, the country’s degree of technological innovation may not be sufficient to obtain renewable energy sources for use in production and industry. To overcome this environmental issue caused by economic activities, innovation in green technology and institutional development are needed. A high level of institutional quality can stimulate technological developments and offset the adverse effects of economic activities on environmental quality. Innovations in green technology can increase energy efficiency and the utilization of renewable energy sources in economic processes, hence reducing carbon emissions. Because of this, the purpose of our study is to investigate the impact that economic growth, consumption of renewable and nonrenewable energy, technological advancement, and institutional quality have on carbon dioxide emissions in the nations that served as our sample. According to the findings, the progression of technology, the utilization of fossil fuels as a source of energy, and the expansion of economic activity all contribute to a rise in carbon dioxide emissions and a decline in environmental quality. The use of renewable energy sources, the establishment of the rule of law, and the maturation of institutions all contribute considerably to an improvement in environmental quality. Both the Environmental Kuznets curve and the Innovation Claudia curve are valid when applied to countries along the Belt and Road.

### Recommendations

As a result, the findings of this study provide evidence that developments in technology, improvements in institutional quality, and the utilization of renewable energy sources are all important factors in attaining sustainable growth and enhancing the quality of the environment. Based on the findings, the countries that were examined should probably make the development of environmentally friendly technologies and renewable energy sources a top priority. An increase in technological innovation will assist the purchase of renewable energy and the enhancement of energy efficiency, hence improving environmental quality. In addition, the results stimulate the use of renewable energy for economic activities and discourage the use of fossil fuels. If the rate of technological progress accelerates, this is achievable. However, strong institutions are necessary for the development of technological advances. Thus, the findings highlight the significance of enhancing institutional quality, as institutional quality can improve technological advances, strengthen other sectors’ contributions to green growth and development, and promote policies for green growth. Consequently, changes in these areas will aid in achieving sustainable development and a higher environmental quality standard.

### Limitation and future research

This study used panel data from Belt and Road countries and explore the effect of institutional quality and technological innovations on carbon dioxide emission. The study is limited to sample countries, econometric models and the variable used. There are several proxies used for technological innovations such as patent application residents, patent nonresidents, high technology export, research and development expenditure and so on however the current study used only patent application residents thus the future study may include other proxies for technological innovations to better investigate its role in environmental sustainability. Likewise, the future studies can use six indicators of institutional quality in such investigation. The future studies may also be conducted on other sample of countries which have dissimilar characteristics from the Belt and road initiative countries and achieve policy suggestions for comparison.
